# Emergence of antiphage functions from random sequence libraries reveals mechanisms of gene birth

**DOI:** 10.1073/pnas.2513255122

**Published:** 2025-10-15

**Authors:** Idan Frumkin, Christopher N. Vassallo, Yi Hua Chen, Michael T. Laub

**Affiliations:** ^a^Department of Biology, Massachusetts Institute of Technology, Cambridge, MA 02139; ^b^Shmunis School of Biomedicine and Cancer Research, Faculty of Life Sciences, Tel-Aviv University, Tel Aviv 6997801, Israel; ^c^Division of Biological and Biomedical Sciences, University of Montana, Missoula, MT 59812; ^d^HHMI, Cambridge, MA 02139

**Keywords:** de novo gene birth, antiphage defense, microbial evolution, random sequences, cellular adaptation

## Abstract

De novo gene birth—the emergence of genes from nongenic sequences—drives biological innovation, yet its adaptive potential remains poorly understood. To investigate this issue, we screened libraries of ~100 million short (semi-)random sequences, mimicking early stages of gene birth, for genes that promote *Escherichia coli* survival during phage infection. This selection uncovered thousands of functional genes that confer viral resistance through at least two distinct mechanisms: 1) activation of a bacterial regulatory system that remodels the outer membrane, which provides broad-spectrum defense, and 2) transcriptional repression of bacterial outer membrane receptors required for phage adsorption, which provides phage-specific protection. Remarkably, unrelated random genes with no sequence similarity produced similar protective phenotypes, revealing that diverse sequences can converge on equivalent functions. We further showed that T4 phage rapidly evolves to counter these novel defenses, acquiring baseplate mutations that enhance adsorption to resistant hosts. Together, these findings demonstrate that random sequences can rapidly evolve into functional genes with direct fitness benefit, highlighting the evolutionary potential of de novo gene birth in the microbial world.

## Significance

How new genes arise and gain function is a central question in biology. New genes can evolve from nongenic DNA, yet their adaptive potential remains unclear. Here, we use millions of (semi-)random sequences as experimental models of emerging genes and find that thousands confer phage resistance in *Escherichia coli*. Expressed random sequences can produce both protein- and RNA-based functions that reprogram cellular systems to counter viral infection. Resistance arises through activation of a cell envelope stress response or downregulation of membrane receptor expression. Our results reveal that genetic novelty, in the form of genes appearing for the first time, can shape host–virus interactions, providing insight into microbial evolution and the surprising ease with which functional genes can emerge.

A hallmark of biology is the natural diversity found at the molecular, cellular, and organismal levels. This diversity demonstrates, and stems from, the ability of organisms to invent, incorporate, and refine new biological functions for adaptation. Although innovation often occurs through gene duplication and divergence (1), new genes can also emerge from previously nongenic loci of (semi-)random sequences, termed de novo gene birth (2).

The de novo birth of new genes has been studied in yeast, insects, plants, and primates through comparative genomics (3, 4), revealing taxonomically restricted “orphans”—genes lacking homologs outside their lineage (5). Synteny-based analyses suggest ~50% of orphan genes are bona fide de novo genes (6). While only few de novo and noncanonical proteins have been studied functionally, some participate in fundamental processes like timing of flowering in *Arabidopsis* (7), male fertility in flies (8, 9), and the proliferation of both healthy (10) and cancerous human cells (11, 12).

In microorganisms, de novo gene birth appears to be a major contributor to genomic diversity (13), with ~600,000 species-specific genes recently identified in gut microbiome samples (14), and over 60,000 predicted gene clusters encoding small proteins of 15 to 70 amino acids found across ~5,600 *Enterobacteriaceae* genomes (15). Most of these clusters show strong lineage specificity and lack detectable homologs, suggesting that the de novo emergence of novel proteins is a common feature in enterobacterial evolution.

Significant gaps remain in our understanding of de novo gene birth. For a nucleotide sequence to gain functionality, it must achieve stable transcription and, to become a protein-coding locus, acquire the necessary signals for translation. To evolve into a mature gene, the nascent protein must also confer a fitness advantage, allowing natural selection to refine it over time (16–18). Recent studies suggest that transcriptional and translational requirements are not major barriers. Genomes exhibit pervasive, rapidly turning-over transcription, continuously exposing nongenic regions to evolutionary testing (18, 19). Many intergenic ORFs are already translated at low levels (10, 20), and large-scale studies indicate that this pervasive translation generates an evolutionarily dynamic pool of small proteins, many of which are noncanonical yet regulated and capable of influencing cellular phenotypes and fitness (21, 22).

Several mechanisms further facilitate gene birth. Divergent promoter architectures can foster bidirectional transcription, seeding new transcripts (23), and experimental evolution demonstrates that promoter recruitment can activate previously silent regions, yielding stably expressed loci (24). Cellular quality-control systems also help stabilize and refine novel loci. Translational errors can expose and purge deleterious sequences through preadapting selection (25), and surveillance pathways degrade faulty translation products from noncoding regions to prevent harmful protein accumulation (26).

Despite widespread transcription and translation of novel sequences, identifying genuine new genes remains challenging due to their lack of evolutionary conservation and the difficulty in distinguishing them from annotation artifacts or transcriptional noise (19, 20, 27). More fundamentally, it remains unclear whether de novo genes can consistently confer fitness advantages and thus be favored by natural selection.

To overcome these challenges, researchers have developed experimental approaches using random nucleotide sequence libraries as controlled models for de novo gene evolution (28–31). These libraries enable systematic sampling of otherwise vast and inaccessible sequence space, but they do not fully capture the genomic context of natural gene birth, which is influenced by nucleotide composition biases and genomic constraints. Nonetheless, because de novo gene birth occurs over evolutionary timescales that are difficult to observe directly, random sequence libraries provide a practical and powerful framework for probing the principles of gene emergence and identifying how functional innovation can originate.

Both random and de novo proteins often contain native-like biophysical features, including defined secondary structures, intrinsic disorder, and solubility profiles comparable to natural proteins. These similarities indicate that structural potential is broadly distributed across sequence space, as even unevolved sequences can adopt soluble, partially folded states (32, 33). In silico evolution experiments further show that stable, globular protein folds can evolve quickly from random sequences under realistic selective pressures, highlighting the value of such libraries as models for uncovering how new, functional protein-coding genes can arise and diversify in nature (34).

Previous in vitro studies have demonstrated the functional potential of such random proteins, showing ATPase activity and diverse binding affinities (28, 35, 36). Random genes have also been shown to confer in vivo fitness benefits in *Escherichia coli*, including antibiotic resistance (37, 38), auxotrophy rescue (39, 40), copper tolerance (41), and toxin inhibition (42). These observations underscore their utility as experimental proxies for understanding how novel genetic elements can gain function and be integrated into cellular systems.

Here, we ask whether random genes can contribute to antiphage defense, one of the strongest selective pressures in bacterial evolution (43). The extensive arsenal of bacterial antiphage defense systems (44, 45) raises a compelling question: can de novo gene birth serve as an evolutionary mechanism for phage resistance? We screened in *E. coli* two libraries of 100 million transcriptionally inducible (semi-)random sequences generated with partially controlled nucleotide composition for defense against phage infection, identifying thousands of such functional sequences that can provide either broad-spectrum or phage-specific protection, primarily through modifying phage adsorption. These (semi-)random genes interfere with viral adsorption through two distinct mechanisms: activation of the Rcs pathway to promote synthesis of a protective extracellular capsule or downregulation of the phage receptor OmpC. Our findings demonstrate how gene emergence can contribute to the fight against viral threats. They offer insights into both the evolution of antiviral systems and the fundamental process of gene birth.

## Results

### Selection for Functional, (Semi-)Random Genes that Provide Antiphage Defense.

**Fig. 1.**
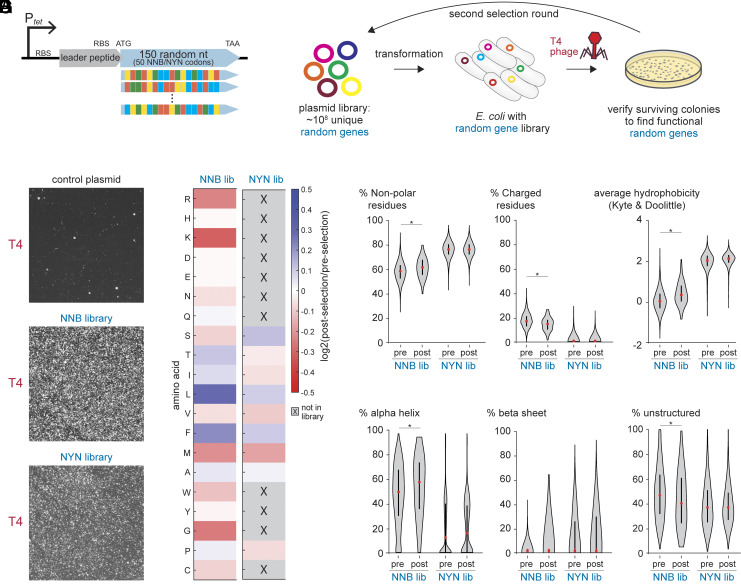
Selection of functional proteins from random sequence libraries that confer antiphage defense. (*A*) Architecture of the random sequence libraries. A tetracycline-inducible promoter (P*_tet_*) drives the expression of a leader ORF followed by an ATG start codon, 150 random nucleotides (50 NNB or NYN codons), a stop codon, and a transcriptional terminator. (*B*) Selection strategy for identifying antiphage random genes. Random sequence libraries of ~10^8^ plasmids were transformed into *E. coli* MG1655 and challenged with T4 phage. Plasmids from survivor colonies were purified, retransformed into naive cells, and reselected to enrich for true hits. (*C*) Bacterial colonies formed in semisolid agar in the presence of T4 phages. NNB and NYN libraries compared to cells harboring a control plasmid expressing only the leader ORF. (*D*) Amino acid frequencies in functional vs. random sequences. ORFs ≥ 30 amino acids from NNB (151 proteins) and NYN (4,385 proteins) libraries postselection were compared with 10,000 randomly sampled ORFs from each library preselection. (*E*) Distribution of nonpolar and charged residues in functional versus randomly sampled sequences from ancestral NNB and NYN libraries. The asterisk indicates Cohen’s d > 0.2. (*F*) Average hydrophobicity comparison (based on Kyte–Doolittle scale) between functional and randomly sampled sequences from NNB and NYN libraries. The asterisk indicates Cohen’s d > 0.2. (*G*) Predicted secondary structure content in functional versus randomly sampled proteins from both libraries. The asterisk indicates Cohen’s d > 0.2.

We sought to identify genes derived from (semi-)random nucleotide sequences that enable bacterial cells to survive phage infection. To this end, we implemented a selection strategy to isolate random genes that confer resistance to the T4 phage, a well-characterized model virus that infects *E. coli* (46). We screened a library of ~100 million plasmids (42), each carrying an inducible promoter driving the expression of two open reading frames. The first ORF was constant and helped reduce variation in translation initiation (47). The second ORF started with an ATG start codon, followed by 50 random NNB codons, and ended with a TAA stop codon (Fig. 1*A*). NNB codons were chosen to keep the amino acid composition similar to the natural genetic code while lowering the chance of early stop codons (*SI Appendix*, Fig. S1).

In parallel, we constructed a second library of comparable size and architecture, containing 50-codon NYN random ORFs. These sequences included only nine possible amino acids, with a compositional bias toward hydrophobic residues (*SI Appendix*, Fig. S1). Deep sequencing of the NNB and NYN ancestral libraries demonstrated their high complexities (*SI Appendix*, Fig. S2).

Our selection approach builds on a previously established screening protocol for identifying natural antiphage defense systems using plasmid libraries(48) (Fig. 1*B*). In this method, *E. coli* MG1655 cells carrying the (semi-)random gene libraries are mixed with T4 phages in a structured medium (soft agar) at a phage concentration that allows individual clones to proliferate into microcolonies before encountering a phage particle. Clones expressing random genes that confer resistance survive the infection and form visible colonies. Unlike liquid-based selection methods, where the strongest resistance phenotypes rapidly dominate, this solid-medium approach enables the detection of a broader spectrum of defense mechanisms, including those with intermediate levels of antiviral protection.

Cells carrying a control vector expressing only the library leader ORF showed few surviving colonies after T4 infection, likely due to previously documented chromosomal mutations conferring T4 resistance (49). In contrast, the NNB and NYN library plates yielded thousands of colonies (Fig. 1*C*). To identify genuine antiphage defense genes of random origin, we purified plasmids from surviving colonies, transformed them into naive *E. coli* MG1655 cells, and performed a second round of selection against the T4 phage. This second selection step minimized false positives arising from chromosomal mutations (42).

We then purified and deep-sequenced NNB and NYN plasmid libraries from cells that survived both rounds of phage selection. We defined functional sequences as those present in the postselection populations at a frequency above 5 × 10^−5^, which corresponded to at least 146 reads per functional sequence in the NNB library and 144 reads per functional sequence in the NYN library (*SI Appendix*, Fig. S2). This analysis identified 358 functional random genes in the NNB library and 4,516 in the NYN library.

### Evolved Random Proteins Exhibit Increased Order and Hydrophobicity.

To characterize the functional random genes involved in antiphage defense, we analyzed ORFs encoding proteins with ≥30 amino acids from positive clones, which included 151 NNB-derived proteins and 4,385 NYN-derived proteins, and compared them to ~10,000 randomly selected ORFs from each respective ancestral library. We pooled all protein sequences within each condition (postselection and preselection), keeping the NNB and NYN libraries separate, and analyzed the global amino acid composition of each of these four pools. This analysis revealed that postselection NNB proteins were enriched in hydrophobic residues, particularly leucine and phenylalanine, and depleted in positively charged residues such as arginine and lysine (Fig. 1*D*). The NYN library exhibited more modest compositional shifts between pre- and postselection pools, likely reflecting its inherent hydrophobic bias introduced by codon design.

Analysis of the composition of individual proteins confirmed the trends observed in the pooled data. Postselection NNB proteins were enriched for nonpolar residues (Cohen’s size *d* = 0.25, *t* test *P* = 0.002) and depleted in charged residues (*d* = 0.28, *P* = 7.4 × 10^−4^) (Fig. 1*E*), resulting in an overall increase in average hydrophobicity (*d* = 0.36, *P* = 1 × 10^−5^) (Fig. 1*F*). Notably, a small proportion (~5%) of NYN variants with minor indels that disrupt the intended codon pattern account for the observed low level of charged residues in both pre- and postselection pools. However, the similar distribution of these variants in both conditions suggests that this synthesis error does not affect our analyses (Fig. 1 *E* and *F*, NYN nonpolar: *d* = 0.04, *P* = 0.02; charged: *d* = 0.07, *P* = 3.9 × 10^−13^, hydrophobicity: *d* = 0.08, *P* = 1.5 × 10^−6^).

To assess how these compositional changes may influence structure, we used NetSurf3.0 (50) to predict residue-level secondary structure probabilities. The results revealed a higher predicted α-helical content (*d* = 0.22, *P* = 0.007) and fewer unstructured regions (*d* = 0.20, *P* = 0.01) in postselection NNB proteins (Fig. 1*G*). β-sheet content remained unchanged (*d* = 0.02, *P* = 0.78), suggesting that the structural bias primarily favored helical organization.

In contrast to the NNB-based proteins, functional NYN proteins showed minimal differences compared to their ancestral sequences (*d* < 0.1 for all metrics) (Fig. 1*G*). We concluded that phage-defensive proteins from the NNB library evolved toward more hydrophobic and structured sequences, whereas the NYN library’s inherent hydrophobicity and structural bias likely contributed to its higher yield of functional genes without requiring further compositional shifts.

### Defensive Random Proteins Block Phage Adsorption By Activating the Rcs Pathway.

**Fig. 2.**
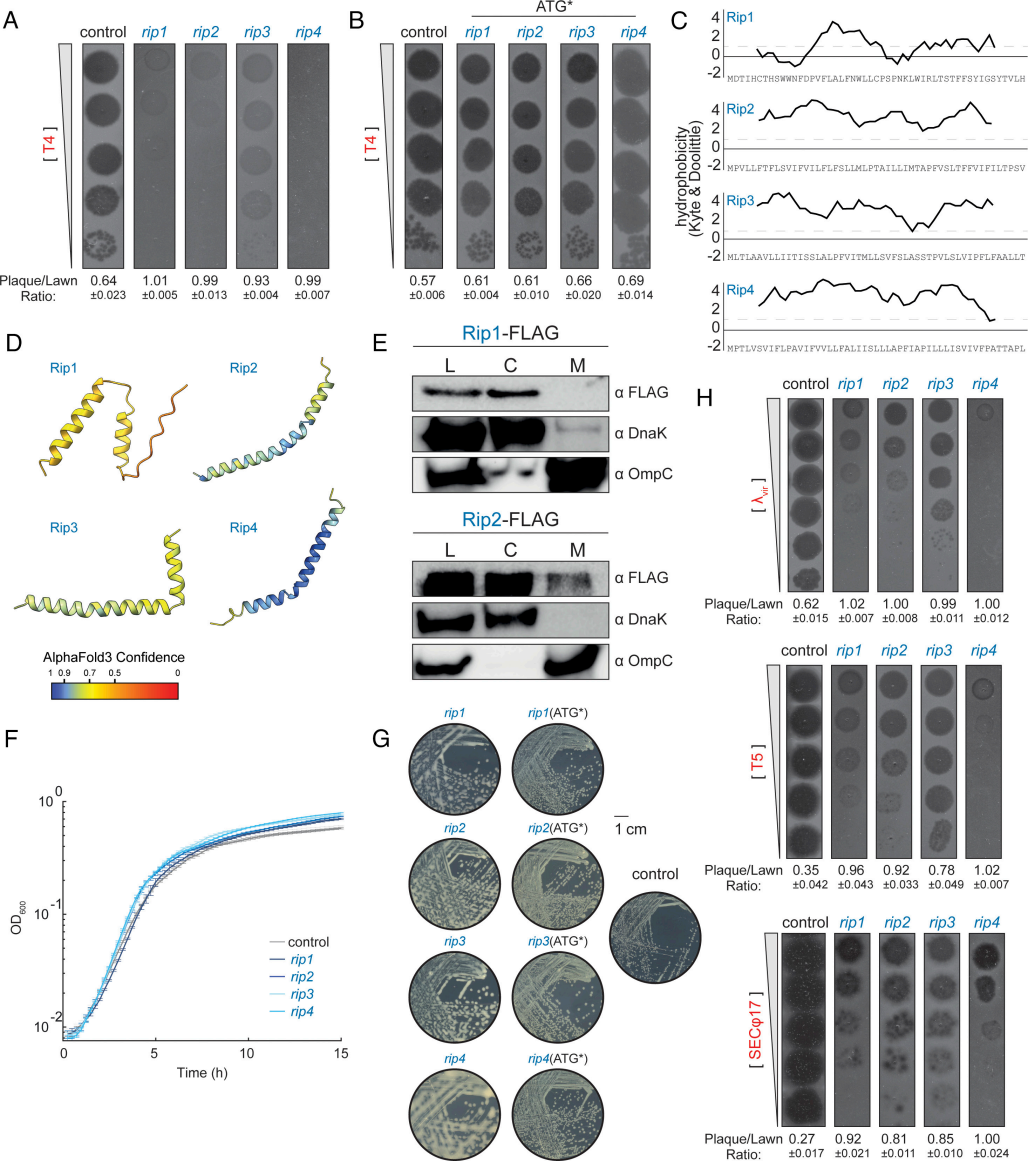
Novel random proteins Rip1-4 induce mucoidy and provide broad-spectrum antiphage defense. (*A*) Phage plaque assay using 10-fold serial dilutions of T4 phages, spotted on lawns of *E. coli* strains expressing *rip1-4* or a control plasmid. Plaque/Lawn ratio: quantitative measurement of antiphage defense, calculated as plaque intensity divided by surrounding lawn intensity. Reported values include the SD calculated from three plaques. This metric is used throughout the study. (*B*) Same as in (*A*) except *rip1-4* plasmids contain start codon mutations. (*C*) Hydrophobicity profiles of Rip1-4 based on the Kyte–Doolittle scale. The dashed line marks regions with values greater than 1, indicating moderate hydrophobicity; values above 1.6 are considered strongly hydrophobic. (*D*) Predicted structures of Rip1-4 generated by AlphaFold3. Color represents the pLDDT score. (*E*) Cellular fractionation of FLAG-tagged Rip1 and Rip2. Anti-FLAG immunoblots were performed on cytosolic and membrane fractions; GroEL and OmpA served as fractionation controls. L = lysate before fractionation, C = cytosolic fraction, M = membrane fraction. (*F*) Growth curves (OD_600_) of cells expressing *rip1-4* or a control plasmid at 30 °C. Values represent means of 12 replicates. (*G*) Colony morphology of cells expressing *rip1-4* or control plasmids grown on solid media. Expression of Rip proteins induces a mucoid phenotype, while cells expressing *rip1-4* with mutated start codons display normal colony morphology. (*H*) Phage plaque assay of λ*_vir_*, T5, or SECφ17 using 10-fold serial dilutions of phages spotted on lawns of *E. coli* strains expressing *rip1-4* or a control plasmid.

We arbitrarily chose four Random Inhibitors of Phage infection (*rip*), transformed them into fresh *E. coli* MG1655 cells, and confirmed that they each reduced T4 infection efficiency in plaque assays (Fig. 2*A*). Mutations in the start codons of *rip1-4* led to the loss of the defense phenotype (Fig. 2*B*), indicating that the protein products of *rip1-4*, not RNAs, were responsible for these phenotypes. As a quantitative measure of antiphage defense, we report a plaque-to-lawn ratio which measures the translucence of phage plaques as a metric of phage productivity (*Materials and Methods*). This approach is used consistently throughout the study and is particularly useful in cases when plaque counts remain close to control levels, but defense can still be detected through changes in plaque opacity.

Sequence analysis revealed that each *rip* gene encodes a 51-amino-acid protein with a hydrophobic profile (Fig. 2*C*). Structure predictions using AlphaFold3 (51) indicated that α-helices are likely the dominant secondary structures in these proteins (Fig. 2*D*). Although their predicted hydrophobic, helical nature suggested potential membrane localization, fractionating cells producing functional FLAG-tagged Rip1 and Rip2 (*SI Appendix*, Fig. S3) revealed that both proteins are in the cytosol (Fig. 2*E*). Rip3 and Rip4 lost function upon tagging (*SI Appendix*, Fig. S3), preventing reliable localization analysis.

To explore the mechanism underlying Rip1-4 antiphage activity, we first asked whether these random proteins affect bacterial growth, as reported for other functional random proteins (37, 40, 42). We found that exponential growth was unaffected (Fig. 2*F*), suggesting that newly emerged proteins like Rip1-4 can integrate into cellular systems without compromising growth. Interestingly, cells producing Rip1-4 reached higher optical densities in the stationary phase, possibly due to accumulated colanic acid secretion from Rcs-pathway activation (see below), which may alter cellular physiology and the OD-to-cell relationship. Although they did not affect growth, producing each of the four Rip proteins resulted in mucoid colonies (Fig. 2*G*), a phenotype associated with colanic acid secretion in *E. coli* (52, 53). Cells expressing *rip1-4* with start codon mutations showed normal colony morphology, confirming that the mucoidy phenotype depends on Rip protein, not RNA (Fig. 2*G*). Because colanic acid secretion provides defense against multiple phage species (54, 55), we tested *rip1-4* against λ, SECφ17, and T5 phages (Fig. 2*H*), which represent viral families distinct from T4 and from each other. The random genes provided protection against each phage despite not being selected on them, suggesting that *rip1-4* each interfere with phage infection through a broad-spectrum mechanism.

**Fig. 3.**
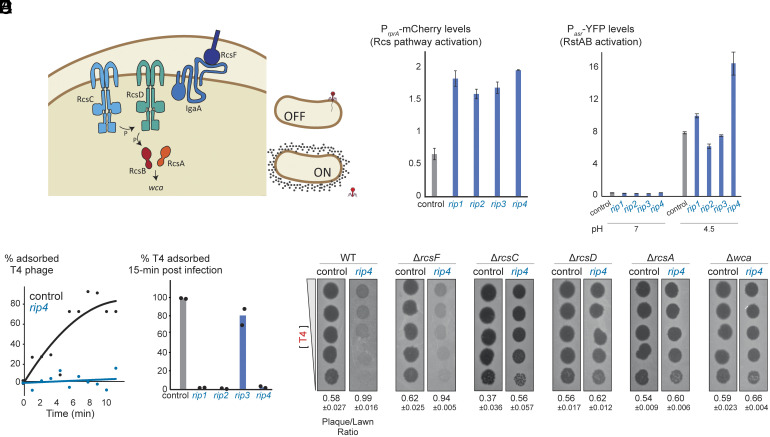
Random proteins activate the Rcs pathway to prevent phage adsorption. (*A*) Cartoon representation of the Rcs stress response pathway in *E. coli* (adapted from ref. 53). A membrane stress signal is detected by RcsF, prompting RcsD to phosphorylate the response regulator RcsB. Phosphorylated RcsB dimerizes with RcsA to activate transcription of the *wca* operon, resulting in capsular polysaccharide synthesis and secretion. (*B*) Rcs pathway activity measured by P*_rprA_*-mCherry fluorescence in strains expressing *rip1-4* or a control plasmid. Values represent means of three biological replicates (*t* test, *P* = 0.04, 0.04, 0.03, and 0.01 for *rip1-4* compared to control, respectively). (*C*) RstAB pathway activity measured by fluorescence reporter at pH 7 and pH 4 in strains expressing *rip1-4* or a control plasmid. Values represent means of three biological replicates. (*D*) Percentage of adsorbed T4 phage remaining in medium over time, comparing adsorption rates between strains expressing *rip4* and a control plasmid. (*E*) Percentage of adsorbed T4 phage in medium 15 min postinfection, comparing strains expressing *rip1-4* versus a control plasmid. Data are the mean of two biological repeats; each black dot is an individual measurement. (*F*) Phage plaque assay of T4 using 10-fold serial dilutions on WT, Δ*rcsF*, Δ*rcsD*, Δ*rcsA*, or Δ*wca* strains expressing *rip4* or a control plasmid.

To understand the molecular basis of this broad protection, we investigated the Rcs stress response pathway, which regulates the *E. coli wca* operon responsible for colanic acid synthesis and secretion during membrane stress (56). This pathway transmits signals from the surface receptor RcsF to the inner-membrane histidine kinase RcsC, which autophosphorylates and transfers phosphate through RcsD to the cytoplasmic response regulator RcsB. RcsB then heterodimerizes with RcsA to activate *wca* operon transcription (Fig. 3*A*). Because Rcs activation can inhibit phage infection of bacterial cells (54), we hypothesized that Rip1-4 production activates the Rcs pathway, explaining our observations.

To test Rcs activation by Rip1-4, we used a fluorescent reporter (mCherry driven by the P*_rprA_* promoter) known to respond to Rcs pathway activity (57). Cells producing any of Rip1-4 showed two- to threefold higher mCherry levels compared to those with a control vector (Fig. 3*B*). We also tested whether Rip1-4 affect other cell envelope stress pathways by examining activity of the RstAB two-component signaling system, which is activated at low pH (58). None of Rip1-4 activated this system at pH 7. At pH 4, Rip1-3 did not interfere with RstAB activation, with Rip4 leading to only a modest ~twofold increase in activity (Fig. 3*C*). Thus, Rip1-4 do not broadly interfere with other cellular processes, consistent with the observation that they do not impair bacterial growth.

We next compared T4 phage adsorption between cells expressing Rip4 and control cells. Time-course measurements revealed a significant reduction in phage adsorption to Rip4-producing cells compared to controls (Fig. 3*D*), consistent with activation of the Rcs pathway and upregulation of capsule biosynthesis. To assess the effect across all variants, we measured adsorption at a single time point (15 min postinfection) and found that production of Rip1, Rip2, or Rip4 significantly reduced T4 adsorption relative to control cells (Fig. 3*E*). Rip3 production resulted in only a ~20% decrease in adsorption, consistent with its weaker antiphage defense phenotype relative to the other Rip proteins.

Finally, we found that cells producing Rip1-4 became susceptible to T4 infection in genetic backgrounds lacking key components of the Rcs signaling pathway: *rcsC* and *rcsD* (phosphotransferases), *rcsA* (transcription factor), or *wca* (capsular operon), confirming that Rcs activation is essential for the antiphage defense function of Rip proteins (Fig. 3*F* and *SI Appendix*, Fig. S4). In contrast, Rip1–4 retained antiphage activity in Δ*rcsF* cells, indicating that their activation of the Rcs pathway occurs independently of the RcsF sensor lipoprotein (Fig. 3*F* and *SI Appendix*, Fig. S4), consistent with previously described RcsF-independent activation mechanisms (59).

### Random Genes Can Provide Specific Defense Against the T4 Phage.

**Fig. 4.**
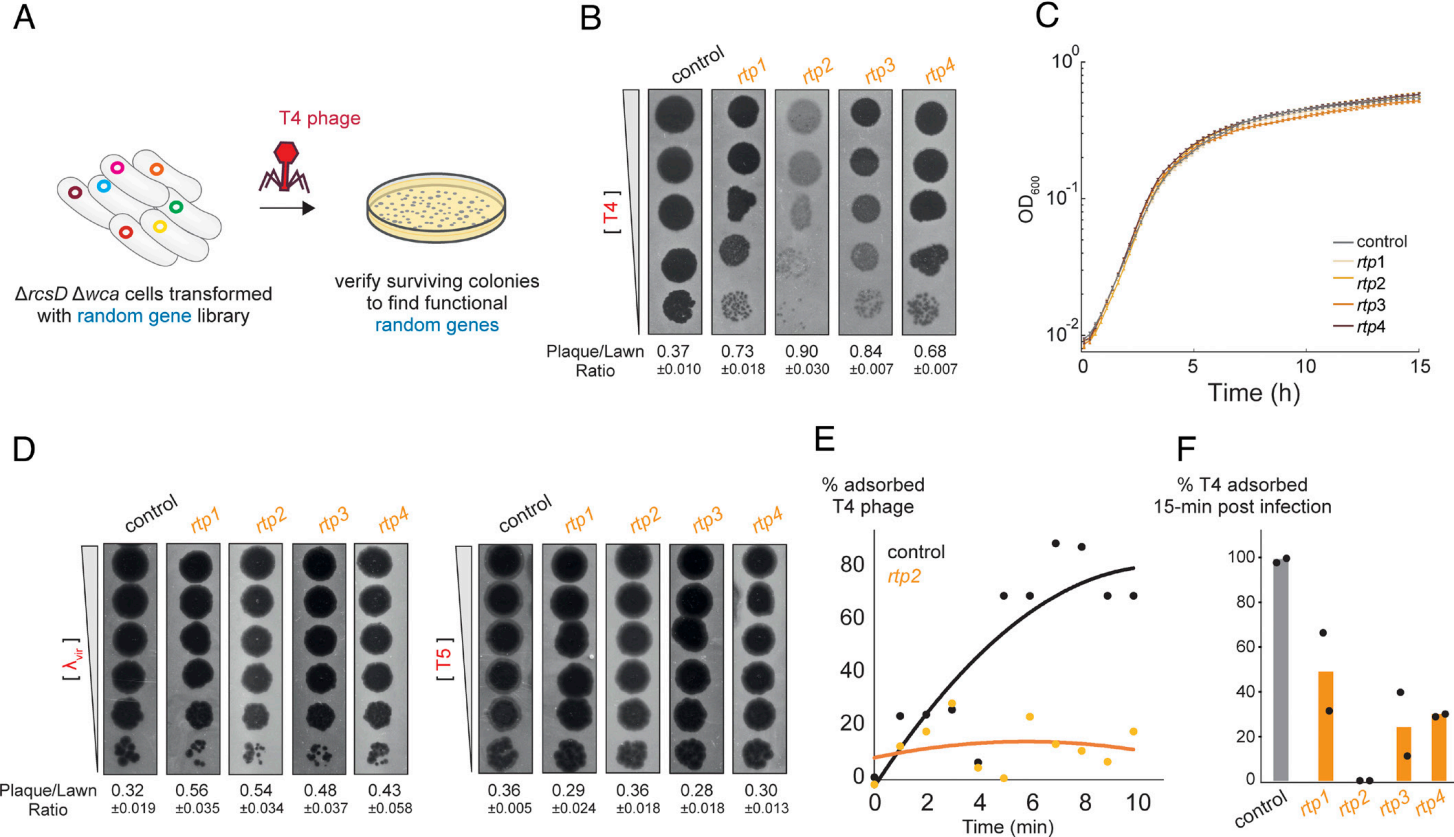
Selection and characterization of random sequences providing T4-specific defense. (*A*) Selecting for T4 -defense genes using NNB and NYN libraries in Δ*rcsD* Δ*wca* strains. (*B*) Phage plaque assay of T4 using 10-fold serial dilutions on Δ*rcsD* Δ*wca* strains expressing *rtp1-4* or a control plasmid. (*C*) Growth curves of Δ*rcsD* Δ*wca* strains expressing *rtp1-4* or a control plasmid at 30 °C. Values represent means of 12 biological replicates. (*D*) Phage plaque assay of λ*_vir_* and T5 using 10-fold serial dilutions on Δ*rcsD* Δ*wca* strains expressing *rtp1-4* or a control plasmid. (*E*) Percentage of adsorbed T4 phages remaining in cell cultures over time, comparing adsorption rates between strains expressing *rtp2* and a control plasmid (measured in parallel with 3D). (*F*) Percentage of adsorbed T4 phage in medium 15 min postinfection, comparing strains expressing *rtp1-4* versus a control plasmid. Data are the mean of two biological replicates; each black dot is an individual measurement.

As our initial screen identified broad-spectrum defenders, we next sought random sequences that could provide phage-specific protection, particularly against T4 phage. To uncover such specialized functional genes, we screened both the NNB and NYN libraries in Δ*rcsD* Δ*wca* cells (Fig. 4*A*). This genetic background prevents Rcs pathway activation and the production of capsular polysaccharides. We reasoned that any protective genes identified in this background would function through Rcs-independent pathways, providing a window into alternative modes of phage resistance.

This screen yielded four random genes altogether—termed Random T4 Inhibitor Products (*rtp1-4*)—that reduced T4 infection efficiency, as evidenced by the formation of cloudier plaques (Fig. 4*B*). All subsequent experiments characterizing *rtp*1-4 were also performed in the Δ*rcsD* Δ*wca* background to ensure consistency with the screening conditions. Expression of *rtp1-4* had minimal to no effect on host cell growth during either exponential or stationary phases (Fig. 4*C*). In contrast to the Rcs-dependent *rip* genes, *rtp1-4* did not confer protection against λ*_vir_* or T5 phages (Fig. 4*D*), indicating specificity for T4. Furthermore, *rtp1-4* expression substantially impaired T4 adsorption to *E. coli* cells (Fig. 4 *E* and *F*), suggesting that interference with adsorption may underlie their protective effect.

In these random sequences, there is potential for translational start from the designed start codon, but also from start codons that occur later in the sequence. We analyzed the nucleotide sequence of the *rtp* genes for functional ORFs, which revealed surprising results. In *rtp2*, the random gene with the strongest anti-T4 phenotype, we identified three possible ORFs (<30 amino acids) starting with ATG or the alternative codons TTG and GTG (*SI Appendix*, Fig. S5*A*). Mutating these start codons individually did not impair the gene’s protective function (*SI Appendix*, Fig. S5*A*), suggesting that *rtp2* may impart its function in the form of RNA. In support of this notion, introducing synonymous mutations (9 and 26 nucleotide substitutions) in the two 5′-proximal ORFs disrupted functionality, whereas synonymous recoding of the 3′-proximal ORF (14 substitutions) preserved function (*SI Appendix*, Fig. S5*A*). Consistently, deleting the 3′ third of *rtp2* did not impair its antiphage activity (*SI Appendix*, Fig. S5*B*). These findings suggest that *rtp2* functions through an RNA product, with its 5′ region being essential for its activity. Similar analyses of *rtp4* revealed that it also acts at the RNA level (*SI Appendix*, Fig. S5*C*). In contrast, *rtp1* remained functional after mutating both possible start codons and applying synonymous recoding, leaving its functional molecule unresolved. This may reflect limitations of synonymous recoding, as RNA-based functions can depend on nucleotide features that cannot be altered without disrupting the coding sequence. For *rtp3*, start-codon mutation preserved activity, but expression of a recoded variant was toxic to cells, leading to inconclusive results (*SI Appendix*, Fig. S5*C*).

### Random Genes Provide T4-Specific Defense Through OmpC Receptor Downregulation.

**Fig. 5.**
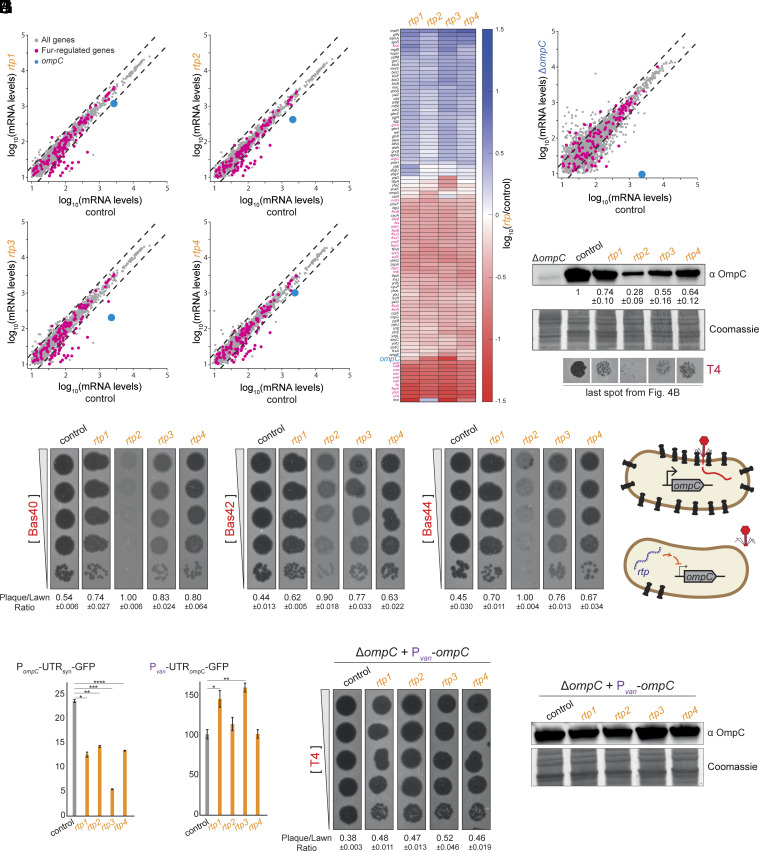
Random genes provide antiphage defense by downregulating the phage receptor OmpC. (*A*) Log_10_ of mRNA levels in transcripts per million (TPM) for *E. coli* genes in strains expressing *rtp1-4* for 5 h compared to control plasmid. Highlighted genes include *ompC*, *trp* operon, and Fur regulon members. (*B*) Heatmap showing log_10_ fold-change in gene expression for all genes that are up- or down-regulated by at least twofold in strains expressing *rtp1-4* compared to control. (*C*) Log_10_ of mRNA levels in TPM for *E. coli* genes in WT vs. Δ*ompC* strains. Data taken from ref. 60. (*D*) Immunoblot of OmpC from strains expressing *rtp1-4* or control plasmid. Coomassie staining served as loading control. Quantification is the mean of n = 3 biological repeats, and values are normalized to levels in the empty vector strain. Plaques are taken from Fig. 4*B*. (*E*) Phage plaque assay of OmpC-dependent phages (Bas40, Bas42, and Bas44) using 10-fold serial dilutions on lawns of strains expressing *rtp1-4* or control plasmid. (*F*) Model of antiphage defense mechanism: *rtp1-4* reduce *ompC* transcription, leading to decreased phage receptor availability and protection against OmpC-dependent phages. (*G*) Fluorescence levels of P*_ompC_*-UTR*_syn_*-GFP reporter in strains expressing *rtp1-4* or a control plasmid. Values represent means of three biological replicates. *P = 0.001, **P = 0.001, ***P = 8.8 × 10^−5^, ****P = 0.0008, *t* test. (*H*) Fluorescence levels of P*_van_*-UTR*_ompC_*-GFP reporter in strains expressing *rtp1–4* or a control plasmid. Values represent means of three biological replicates. *P = 0.003, **P = 0.0002, *t* test. (*I*) Phage plaque assay of T4 using 10-fold serial dilutions on a lawn of Δ*ompC* strains expressing *rtp1-4* or a control plasmid, with OmpC production driven by a vanillate-inducible promoter. (*J*) Immunoblot of OmpC in Δ*ompC* strains expressing *rtp1-4* or a control plasmid, with OmpC production driven by a vanillate-inducible promoter. Coomassie staining served as loading control.

To investigate how random genes confer defense specifically against T4 phage infection, we used RNA-sequencing to examine the transcriptomes of *E. coli* cells expressing *rtp1-4*. Despite having nucleotide sequences with no detectable similarity (*SI Appendix*, Fig. S6), all four *rtp* genes induced modest but comparable global transcriptional changes (Fig. 5 *A* and *B*). Comparative analysis of fold-changes relative to control cells revealed highly correlated transcriptional profiles between the four *rtp*-expressing strains (*SI Appendix*, Fig. S7), suggesting a common cellular response. Each strain exhibited a relatively small number of differentially expressed genes exceeding a twofold threshold: 16, 4, 33, and 17 genes were upregulated, while 33, 42, 53, and 38 genes were downregulated in cells expressing *rtp1-4*, respectively (Fig. 5*B*). Among the affected genes, the only group of genes with a shared function that we detected was the Fur regulon, which responds to cellular iron homeostasis, with several of its members showing downregulation. The specific importance of this observation remains unknown.

Importantly, *ompC* transcript levels were reduced to 55, 20, 9, and 50% of control levels in *rtp1-4*-expressing cells, respectively (Fig. 5*A*). In contrast to the relatively specific transcriptional changes induced by *rtp* gene expression, Δ*ompC* cells exhibited broad transcriptional remodeling, with 250 genes upregulated and 324 genes downregulated at a twofold threshold (60) (Fig. 5*C*). Because OmpC serves as the primary receptor for T4 phage adsorption and deletion of *ompC* confers resistance to T4 (61), we hypothesized that *rtp*-mediated downregulation of *ompC* reduces T4 phage susceptibility by limiting receptor availability. Supporting this model, and consistent with the RNA-seq findings, OmpC protein levels were also reduced in these strains, with the degree of reduction correlating with the strength of T4 protection (Fig. 5*D*).

*rtp1-4* did not protect against phages that do not use OmpC as a receptor (Fig. 4*D*). However, when we tested three OmpC-dependent phages from the BASEL collection (62)—Bas40, Bas42, and Bas44—strains expressing *rtp1-4* showed reduced infection efficiency compared to controls (Fig. 5*E*). Notably, *rtp2* and *rtp3* provided the strongest protection, consistent with their superior performance against T4 (Fig. 4*B*).

We also confirmed that the *rtp2* mutations tested against T4 in *SI Appendix*, Fig. S5*A* showed similar effects against Bas40, providing further evidence for RNA-level activity rather than protein-mediated defense for this random gene (*SI Appendix*, Fig. S5*D*).

Taken all together, our results support a model (Fig. 5*F*) in which *rtp* genes confer specific antiphage defense by downregulating *ompC* expression, thereby reducing the availability of the primary receptor required for infection.

### *rtp1-4* Reduce *ompC* Expression Via Transcriptional Repression.

The expression of *ompC* in *E. coli* is regulated at multiple levels. At the transcriptional level, it is activated by phosphorylated OmpR, the response regulator of the EnvZ-OmpR two-component system, which responds to changes in osmolarity (63). Posttranscriptionally, *ompC* translation is regulated by several small RNAs (sRNAs), including *micC* and *rybB*, which bind to the 5′ untranslated region (UTR) of *ompC* mRNA in a manner dependent on the RNA chaperone Hfq to block translation initiation (64).

We investigated whether *rtp1-4* regulate *ompC* at the transcriptional or posttranscriptional level. First, we examined whether *rtp1-4* activity depends on Hfq. However, all four genes retained functionality in *Δhfq* cells (*SI Appendix*, Fig. S8). In addition, sequence analysis revealed no substantial similarity between *rtp1-4* and known *ompC*-regulating sRNAs (*SI Appendix*, Fig. S9), making an sRNA mimicry mechanism unlikely.

To further distinguish between transcriptional and posttranscriptional regulation, we tested a GFP reporter construct driven by the native *ompC* promoter and a synthetic 5′ UTR insensitive to sRNA-mediated repression. Expression of each *rtp* gene significantly reduced GFP fluorescence by ~40-80%, suggesting that *rtp1-4* act at the level of *ompC* promoter activity (Fig. 5*G*). In contrast, when GFP was driven by a vanillate-inducible promoter coupled to the native *ompC* 5′ UTR, *rtp1* and *rtp3* expression led to a ~50% increase in fluorescence, and *rtp2* and *rtp4* had no statistically significant effect. These results further support a transcription-modulating mechanism of regulation, rather than posttranscriptional control via sRNA interactions with the 5′ UTR (Fig. 5*H*).

Consistent with this conclusion, expressing *ompC* from a vanillate-inducible promoter in *ΔompC* cells substantially reduced the protective effect of *rtp1-4* (Fig. 5*I*) and prevented downregulation of OmpC protein levels (Fig. 5*J*). Together, these findings demonstrate that *rtp1-4* reduce OmpC levels by diminishing *ompC* transcription, thereby reducing phage adsorption and conferring anti-T4 defense. To assess whether this regulation depends on the global transcriptional regulator OmpR, we tested the *rtp* genes in Δ*ompR* cells (*SI Appendix*, Fig. S10). Only *rtp3* showed a notably weaker phenotype in this background, suggesting that *rtp3* may act through an OmpR-dependent mechanism, whereas the regulatory effects of *rtp1*, *rtp2*, and *rtp4* appear OmpR-independent.

### Phage T4 Can Evolve Resistance to *rtp*-Mediated Defense.

**Fig. 6.**
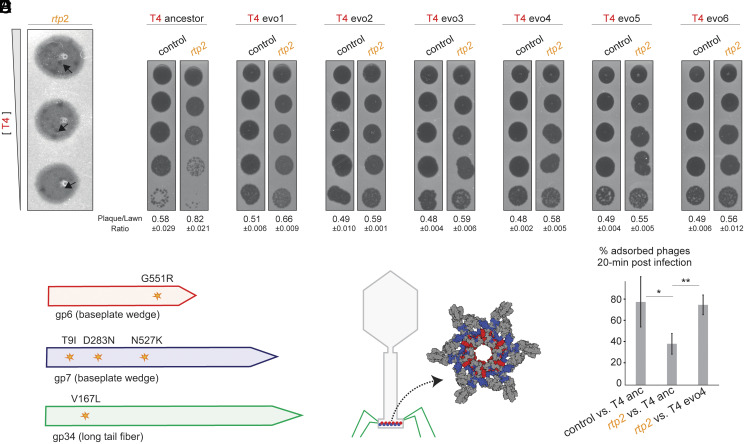
T4 phage evolves resistance to *rtp2*-mediated defense through baseplate mutations that enhance adsorption. (*A*) Plaque morphology of T4 phage plated on lawns of *E. coli* expressing *rtp2*. Emergence of small, clear plaques within the resistant lawn, marked with arrows, indicates evolved phage variants. (*B*) Phage plaque assay of ancestral or evolved T4 phages using 10-fold serial dilutions on a lawn of strains expressing *rtp2* or a control plasmid. (*C*) Schematic of the T4 tail structure highlighting Gp6, Gp7, and Gp34, which are involved in host receptor recognition and infection initiation. (*D*) Schematic of mutations identified in evolved T4 genomes. Five of six isolates carried mutations in gp6, gp7, or gp34. (*E*) Percentage of adsorbed ancestral and evolved T4 phages remaining in cell cultures over time, comparing adsorption rates between strains expressing *rtp2* and a control plasmid. Values represent means of four biological replicates. *P = 0.03, **P = 0.002, *t* test.

When plating T4 phage on *E. coli* cells expressing *rtp2*, we observed the emergence of small, more strongly cleared zones within the otherwise resistant lawn (Fig. 6*A*). We hypothesized that these areas contained mutant phages capable of more effectively infecting *rtp2*-expressing cells, potentially by overcoming the reduction in OmpC levels. To test this possibility, we isolated six such phage clones and measured their infection efficiency on *rtp2*-expressing cells. All six exhibited an ~10-fold increase in infection efficiency compared to the ancestral T4 used in our experiments (Fig. 6*B*).

To investigate the genetic basis of this adaptation, we performed deep sequencing of the mutant phage genomes and identified between 1 and 7 mutations per isolate. Strikingly, five of the six evolved phages carried mutations in one of three genes: *gp6*, *gp7*, or *gp34*, all of which encode essential structural components of the baseplate and long tail fibers (Fig. 6 *C* and *D* and Dataset S2). These proteins are critical for host recognition and infection initiation: Gp34 anchors the long tail fibers to the baseplate and mediates contact with the host receptor OmpC, while Gp6 and Gp7 form part of the baseplate wedge and transmit the mechanical signal that triggers tail sheath contraction upon receptor binding (65).

The repeated appearance of mutations in baseplate-associated genes, together with our observation that T4 adsorption is reduced in *rtp2*-expressing cells, led us to hypothesize that the evolved phages had enhanced adsorption capabilities. To test this directly, we focused on a representative evolved strain, T4 evo4, which carries only a single point mutation in *gp7*. Adsorption assays confirmed that T4 evo4 exhibited improved attachment to *rtp2*-expressing cells compared to the ancestral T4 phage (Fig. 6*E*).

We also compared T4 adsorption rates in control cells and *rtp3*-expressing cells (*SI Appendix*, Fig. S11). Notably, we found no significant difference between ancestral and evo4 phage in control cells, suggesting that the mutation did not confer a general fitness cost or benefit in the original host context. Despite being isolated on *rtp2*-expressing cells, evo4 also showed improved adsorption on *rtp3*-expressing cells. This observation supports the idea that *rtp2* and *rtp3* confer antiphage defense through a similar mechanism of reducing OmpC levels, which evo4 can overcome.

These results demonstrate that T4 phage can rapidly evolve to overcome *rtp2*-mediated defense by acquiring mutations that enhance adsorption efficiency. This likely occurs through fine-tuning of baseplate dynamics and receptor engagement, underscoring the evolutionary plasticity of phage infection mechanisms in response to host-imposed constraints.

## Discussion

This work demonstrates how de novo gene birth can provide immediate benefits to bacteria under viral threat. By screening two libraries comprising ~100 million (semi-)random sequences each, we identified over 4,500 distinct random genes that confer protection against phage infection. These findings highlight the capacity of unevolved sequences to give rise to biologically useful functions, underscoring a central principle in evolutionary biology: Functional novelty can arise not only through gene duplication and divergence but also directly from nongenic DNA.

The immense diversity of possible protein sequences makes random libraries a powerful experimental proxy for studying gene emergence. Even a short 150-nucleotide gene has ~10^90^ possible sequences, vastly exceeding the ~10^40^ cells estimated to have ever existed (66). This creates an apparent paradox: If functional sequences are exceedingly rare, de novo gene birth should be nearly impossible. However, the widespread presence of orphan genes across all branches of life—and our own observation of thousands of functional (semi-)random genes—suggests that functional sequences may be much more common than traditionally assumed, supporting plausible mechanisms for the de novo birth of new genes.

By systematically exploring random sequences, we can empirically test how frequently functional genes arise and what roles they can perform, illuminating the earliest stages of gene birth. The two libraries used here—NNB and NYN—differ in their nucleotide composition, with the NYN library enriched for hydrophobic residue codons. This enrichment likely contributes to its ~10-fold higher hit rate under identical screening conditions, as hydrophobic amino acids appear advantageous in the context of our assay. From these libraries, we recovered ~350 and ~4,500 functional genes, respectively, with hit rates consistent with our previous work identifying ~2,000 random sequences that inhibit the MazF toxin using the NNB library (42).

How likely are genes similar to our (semi-)random hits to emerge naturally? Previous studies have shown that naturally occurring de novo proteins in eukaryotes often resemble unevolved random sequences of equivalent length and composition in their structural properties (32). Computational analyses further demonstrate that random sequences derived from DNA with 40 to 60% GC content occupy structural property spaces that overlap with the human proteome (67). Similarly, intergenic ORFs in yeast noncoding regions can produce proteins with a wide range of folding potentials, and comparisons to scrambled controls suggest this diversity comes from basic sequence properties rather than evolutionary fine-tuning (68). Together, these lines of evidence suggest that the functional capacity observed in our screen of (semi-)random libraries may reflect an evolutionary scenario in which previously nongenic sequences acquire function and serve as raw material for gene birth.

We also acknowledge several limitations of using random sequence libraries. They do not fully recapitulate the evolutionary context, including the selective pressures, genomic neighborhoods, and regulatory landscapes that shape natural gene emergence. They sample sequence space uniformly, overlooking the mutational biases and constraints that influence which sequences are accessible in genomes. Finally, while our screen identifies sequences conferring phage resistance under defined laboratory conditions, it does not account for the context-dependent pleiotropic effects that could influence gene fixation in nature.

Intriguingly, the recovered random proteins we followed up on provide a fitness benefit without causing any detectable cost to growth, a distinction from previously reported functional random proteins that often impair growth (37, 42). Here, broad-spectrum protective proteins (Rips) and T4-specific inhibitors (*rtp* genes) preserved normal cellular physiology during exponential growth and sometimes enhanced final yield. These findings underscore the notion that random sequences need not be disruptive. Rather, they can act as subtle regulators, modulating existing cellular networks in ways that are both tolerable and advantageous under selective pressure.

These beneficial effects arise via two distinct mechanisms. First, we found broad-spectrum defense genes that activate the Rcs stress response, leading to colanic acid secretion and mucoidy. This capsule formation physically blocks phage adsorption and delays infection. Second, we identified T4-specific genes that downregulate the outer membrane receptor OmpC to prevent adsorption. By reducing receptor levels rather than deleting the gene altogether, cells may preserve the physiological functions of OmpC under other conditions, thereby avoiding the fitness cost associated with *ompC* deletion. In support of this, transcriptomic analysis revealed that random gene-mediated OmpC reduction did not trigger the broad regulatory shifts seen in ∆*ompC* cells.

Perhaps most striking is that very different random sequences converged on similar phenotypes. Despite lacking sequence similarity, *rtp1-4* all reduced OmpC levels through modest yet consistent transcriptome shifts. This convergence points to a surprisingly permissive cellular system capable of integrating novel regulators into existing networks. Notably, *rtp3* behaves differently in the Δ*ompR* mutant, suggesting that these random genes may achieve similar cellular phenotypes through distinct mechanisms of OmpC down-regulation.

The apparent ease with which random sequences can rewire transcription has profound implications for gene birth and bacterial evolution. Downregulating phage receptors like *ompC* may be more evolutionarily accessible than evolving de novo genes whose products directly combat viruses. Preventing phage entry altogether may offer a more immediately available route to confer protection. This notion is consistent with the use of receptor modifications as antiphage defense mechanisms (69).

Finally, the rapid evolution of T4 phage to overcome *rtp*-mediated defense validates the biological relevance of our synthetic system. Phage mutations in baseplate proteins restored infectivity, mirroring the ancient bacteria–phage arms race where innovation drives counterinnovation. Our findings demonstrate that functional novelty can rapidly emerge from random sequence space, integrating into cellular networks with surprising precision to provide immediate fitness benefits. In the evolutionary arms race between microbes and viruses, even subtle innovations can trigger reciprocal adaptations—revealing how de novo genes might fuel the endless cycle of biological innovation that has shaped life for billions of years.

## Materials and Methods

### Plasmids, Strains, and Growth Conditions.

All strains and plasmids used in this study are listed in Dataset S1. *E. coli* was grown in LB medium (10 g/L NaCl, 10 g/L tryptone, 5 g/L yeast extract). Media for selection or plasmid maintenance were supplemented with carbenicillin (100 μg/mL), chloramphenicol (20 μg/mL), or kanamycin (30 μg/mL) as appropriate. Overnight cultures were prepared in the same medium used in a given experiment and cells were grown at 30 °C and 180 rpm in a tube rotator. The tetracycline- and vanillate-inducible promoters were induced with 100 ng/μL anhydrous tetracycline (aTc) and 20 µM vanillate, respectively. Plasmids were generated by Gibson assembly according to the manufacturer’s protocol.

### Assembly and Transformation of the Random Gene Library.

The NNB library was previously described in detail (42). The NYN library was constructed in parallel using the same methods. Random gene libraries were constructed by cloning 150-nucleotide sequences (50 random codons in which the three nucleotides correspond to sequence with the pattern N-C/T-N) between an ATG start codon and two TAA stop codons into the plasmid vector. After PCR amplification of ssDNA oligos synthesized by IDT (16 cycles, six pooled reactions), 193-nucleotide amplicons were purified and digested with Esp3I. The digested inserts were ligated to Esp3I-cut vector using a cycling ligation protocol (100 cycles of 16 °C/37 °C). Following electroporation into DH10B cells (~10^8^ transformants), the libraries were grown overnight in LB + carbenicillin, with half stored at −80 °C and half used for plasmid preparation. The plasmid libraries were then electroporated into *E. coli*, yielding ~5 × 10^8^ transformants per library.

### Amplicon Sequencing of Random Library and analysis.

To assess the library complexity pre- and postselection, random sequences were amplified using a forward primer that included the Illumina adapters and indices as well as a region directly upstream of the random nucleotides and a reverse primer matching a region immediately downstream of the random nucleotides with adapters and indices. PCRs were performed using KAPA polymerase enzyme (Roche) for 10 amplification cycles according to the manufacturer’s recommendations. Four independent reactions were performed and combined to minimize PCR bias. Amplicons were purified from an agarose gel using a Zymo Gel DNA Recovery kit. Paired-end sequencing was performed on an Illumina MiSeq at the MIT BioMicroCenter.

### Random Sequence Analyses.

Paired-end reads were merged using PEAR (70) with default parameters. Merged reads were processed with cutadapt (71) to remove flanking sequences and isolate the random sequences with their start and stop codons. Sequences were then clustered at 95% identity using VSEARCH (72) cluster_size command. ORF translation was performed using MATLAB’s nt2aa function, amino acid composition was analyzed using aacount, and hydrophobicity was calculated using proteinpropplot. Secondary structure predictions were generated using NetSurfP-3 (50). NNB and NYN ancestral libraries were sequenced in parallel, and NNB data were previously reported (42).

### Screening NNB and NYN Libraries Against T4 Phage.

Screening random gene libraries for phage-defense genes was performed in two rounds. For the first round, NNB library, NYN library, and control vector were grown overnight at 30 °C in LB medium supplemented with antibiotics. Cultures were diluted 1:10 in fresh medium containing anhydrotetracycline (aTc) and grown for 5 h at 30 °C in darkness. Cells were pelleted and resuspended to OD600 = 1.0 (~3 × 10^8^ CFU/mL). For selection, 500 µL of cell suspension was mixed with 100 µL of T4 phage (10^8^ PFU/mL) and spread on LB agar plates containing carbenicillin and aTc. After overnight growth at 30 °C, surviving colonies were collected and plasmids were purified. These plasmids were transformed into fresh *E. coli* MG1655 cells (~5 × 10^7^ transformants per library) for a second round of selection.

### Plaque and Phage Assays.

Bacterial strains were grown overnight at 30 °C in LB medium with antibiotics, diluted 1:10 in fresh medium supplemented with anhydrotetracycline (aTc), and grown for 5 h at 30 °C. Top agar (LB + 0.5% agar) was melted and cooled to 55 °C. Phage stocks were serially diluted 10-fold in LB medium. For plaque assays, 40 µL of bacterial culture was mixed with 4 mL of molten top agar supplemented with carbenicillin and aTc, and poured onto prewarmed LB agar plates containing the same supplements. After 15 min of solidification, 4 µL of each phage dilution was spotted onto the bacterial lawn. Phage spots were left to dry and then incubated overnight at 30 °C in darkness.

### Measuring Plaque/Lawn Ratio Using ImageJ.

To quantitatively assess the level of antiphage defense, we measured the grayscale intensity of a representative plaque in the last spot of each phage plaquing assay and normalized it to the intensity of the surrounding bacterial lawn. In this analysis, a value of 1 indicates strong and near complete defense, where the region of phage spots appears similar in intensity to the surrounding lawn (i.e., no visible clearing). Lower values reflect successful phage infection, corresponding to clearer plaque formation and more effective lysis of the bacterial lawn.

### Phage Adsorption Assay.

Phage adsorption was measured by quantifying free phage particles after incubation with bacterial cells, adapted from ref. 73. Overnight bacterial cultures were diluted 1:100 in LB medium and grown at 30 °C to OD600 ~0.3 to 0.4. Phage was added at a multiplicity of infection (MOI) of 0.1, and cultures were incubated for 20 min at 30 °C. At this timepoint, 500 µL samples were mixed with 200 µL ice-cold chloroform, vortexed for 30 s, and centrifuged at 5,000×*g* for 1 min to remove bacterial cells. Free phage particles in the supernatant were quantified by the plaque assay. Adsorption efficiency was calculated as (PFUcontrol − PFUsample)/PFUcontrol × 100%.

See *SI Appendix* for methods on measurements of bacterial liquid-growth measurements, fluorescence levels with flow cytometry, western blot analysis, RNA extraction and sequencing, and protein structure prediction with AlphaFold3.

## Supplementary Material

Appendix 01 (PDF)

Dataset S01 (XLSX)

Dataset S02 (XLSX)

## Data Availability

RNA-seq data can be found in BioProject accession number PRJNA1251989 (74). All other study data are included in the article and/or supporting information.
