# Supplementary material for: Emergence of antiphage functions from random sequence libraries reveals mechanisms of gene birth
Source: Proc Natl Acad Sci U S A. 2025 Oct 15;122(42):e2513255122. doi: 10.1073/pnas.2513255122 (PMC12557735; doi:10.1073/pnas.2513255122)
Supplement: Supplementary file 1 — Appendix 01 (PDF) [file pnas.2513255122.sapp.pdf]

Supporting Information for
Emergence of anti-phage functions from random sequence libraries reveals mechanisms of gene birth

Idan Frumkin[1,2,#,*], Christopher N. Vassallo[1,#,*], Yi Hua Chen[1], and Michael T. Laub[1,3,*]

Paste Idan Frumkin
Email: idanfrumkin@tauex.tau.ac.il

Paste Christopher N. Vassallo
Email: cvassall@mit.edu

Paste Michael T. Laub
Email: laub@mit.edu

This PDF file includes:

Supporting text
Figures S1 to S11
Legends for Datasets S1 to S2
SI References

Other supporting materials for this manuscript include the following:

Datasets S1 to S2

Supporting Information Text

Supporting Methods

Bacterial liquid-growth measurements

Bacterial strains were grown overnight at 30 °C in LB medium supplemented with 50 µg/mL carbenicillin, diluted 1:50, and grown an additional overnight at 30 °C. The next day cultures were diluted 1:200 in medium supplemented with 100 µg/mL aTc and seeded into a 96-well plate (160 µL culture overlaid with 70 µL mineral oil) such that each culture had 12 replicates on the same plate and plates were replicated independently at least three times. Growth was monitored at 15 min intervals for 15 hours with orbital shaking on a plate reader (Biotek) at 30 °C.

Measurements of fluorescence levels with flow cytometry

Cultures were grown overnight at 30 °C in LB supplemented with appropriate antibiotics. Cultures were diluted 1:500 in medium supplemented with 100 µg/mL aTc to induce expression of the random genes (or a control vector) and grown for 5 hours at 30 °C in darkness. Cultures were then diluted 1:40 into PBS supplemented with 500 µg/mL kanamycin to stop translation, and incubated at room temperature for 10 min. Fluorescence was measured on a Miltenyi MACSQuant VYB. ~30,000 cells were measured per replicate and FlowJo was used to analyze the data, gating on single live cells, and extracting the median of the msfGFP distribution.

Western blot analysis of steady-state OmpC levels

Cultures were grown overnight at 30 °C in an appropriate medium, diluted 1:50 the next day in medium supplemented with 100 ng/µL aTc to induce expression of the random genes, and grown at 30 °C for 5 hours. 1 mL of cells was pelleted and flash-frozen. Pellets were then resuspended in 1× Laemmli sample buffer (Bio-Rad) supplemented with 2.5% β-mercaptoethanol normalized to the OD_{600} of the culture at the moment of collection (100 µL per OD_{600} 1.0). Samples were boiled at 95 °C for 5 min, analyzed by 4%-20% SDS–PAGE, and wet-transferred to a 0.2 µm PVDF membrane by application of 100V for 1 hour. To visualize proteins, an anti-OmpC antibody (ThermoFisher) and horse-radish peroxidase conjugated

goat anti-rabbit secondary antibody (Thermo Fisher) were used at a final concentration of 1:1000. SuperSignal West Femto Maximum Sensitivity Substrate (Invitrogen) was used to develop the blots. Blots were imaged by a ChemiDoc Imaging system (Bio-Rad). Loading controls were performed using a Coomassie stain as previously described(1).

RNA extraction and sequencing

E. coli strains were grown overnight in LB supplemented with appropriate antibiotics at 30 °C. Overnight cultures were diluted 1:50 in 25 mL of medium supplemented with 100 ng/µL aTc to induce expression of the random genes, and grown for 5 hours at 30 ºC. At that time, 1 mL of each culture was mixed with stop solution (110 µL; 95% ethanol and 5% phenol) and pelleted by centrifugation for 30 sec at 16,000 x g on a tabletop centrifuge. Pellets were flash-frozen and stored at -80 °C. Cells were lysed by adding TRIzol (Invitrogen) preheated to 65 °C directly to pellets, followed by 10 min of shaking at 65 °C and 2,000 rpm on a ThermoMixer (Eppendorf). RNA was extracted from the TRIzol mixture using Direct-zol (Zymo) columns according to manufacturer's protocol. Genomic DNA was removed by adding 2 µL of Turbo DNase (Invitrogen) in a 100 µL final volume using the provided buffer and incubating for 30 minutes at 37 °C. DNase reaction products were cleaned up with a Zymo RNA clean and concentrator kit and eluted in 25 µL water. Samples were sent to SeqCenter for library preparation and sequencing. Library preparation was performed using Illumina's Stranded Total RNA Prep Ligation with Ribo-Zero Plus kit and 10bp unique dual indices (UDI). Sequencing was done on a NovaSeq X Plus.

Geneious Prime 2022.2.2 was used to map reads to the *E. coli* MG1655 genome (accession number NC_000913) with default parameters and to calculate Transcripts Per Million (TPM) values for all genes. TMP values of each sample were normalized by the median TMP value of a given sample to make all samples comparable(2). Raw data can be found in BioProject accession number PRJNA1251989.

Protein structure prediction with AlphaFold3

The predicted structures of *rips1-4* complex was generated using AlphaFold3 webserver with default parameters(3).

Supporting Information Text

Supporting Information Appendix, Figures S1 to S11

A

	NNN Codons	NNB Codons	NYN Codons
Ala [A]	4	3	4
Arg [R]	6	4	0
Asn [N]	2	2	0
Asp [D]	2	2	0
Cys [C]	2	2	0
Gln [Q]	2	1	0
Glu [E]	2	1	0
Gly [G]	4	3	0
His [H]	2	2	0
Ile [I]	3	2	3
Leu [L]	6	4	6
Lys [K]	2	1	0
Met [M]	1	1	1
Phe [F]	2	2	2
Pro [P]	4	3	4
Ser [S]	6	5	4
Thr [T]	4	3	4
Trp [W]	1	1	0
Tyr [Y]	2	2	0
Val [V]	4	3	4
Stop	3	1	0
Codons	64	48	32
AA	20	20	9

C

	E. coli genome	NNB	NYN
gg	6.34	4.9	1.24
ga	5.97	4.2	1.48
gt	5.75	7.84	5.18
gc	8.48	5.43	4.1
ag	5.19	4.27	1.49
aa	7.15	3.94	3.99
at	6.52	7.5	8.54
ac	5.34	3.87	5.3
tg	7.32	8.2	5.67
ta	4.48	7.46	8.27
tt	7.05	12.29	15.29
tc	5.51	7.72	10.95
cg	7.69	5	3.61
ca	6.61	3.99	5.58
ct	5.03	8.04	11.16
cc	5.57	5.35	8.15

B

Side Chain	Amino acids	NNN %	NNB %	NYN %
Acidic [-]	Asp, Glu	6.3	6.3	0.0
Basic [+]	Arg, His, Lys	15.6	14.6	0.0
Non-polar aliphatic	Ala, Ile, Leu, Met, Val	28.1	27.1	56.3
Aromatic	Phe, Trp, Tyr	7.8	10.4	6.3
Polar	Asn, Cys, Gln, Ser, Thr	25.0	27.1	25.0
Special Features	Gly, Pro	12.5	12.5	12.5

Figure S1 | Codon usage of NNN, NNB, and NYN genetic codes.

(A) Number of codons for each amino acid in the NNN, NNB, and NYN genetic codes.

(B) Percentage of amino acid groups in the NNN, NNB, and NYN genetic codes.

(C) Dinucleotide frequencies of functional NNB and NYN sequences compared with the *E. coli* genome.

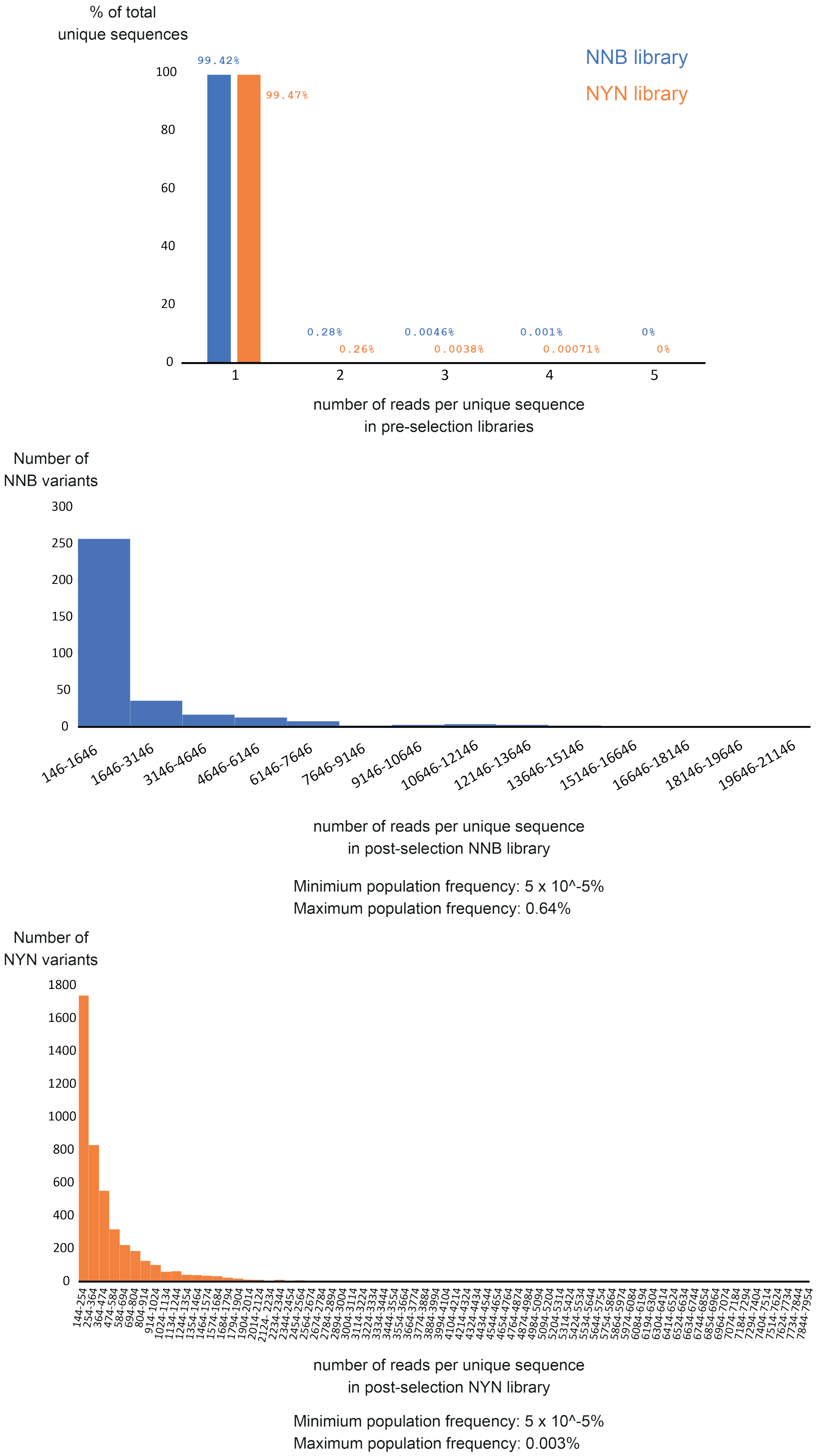

Figure S2 | Read counts in the unselected random gene libraries

Distribution of sequencing read counts per unique sequence in the unselected random gene libraries. Total read count per library was ~300,000. NNB data taken from *Frumkin and Laub, Nat Ecol Evol 7, 2067–2079 (2023)*.

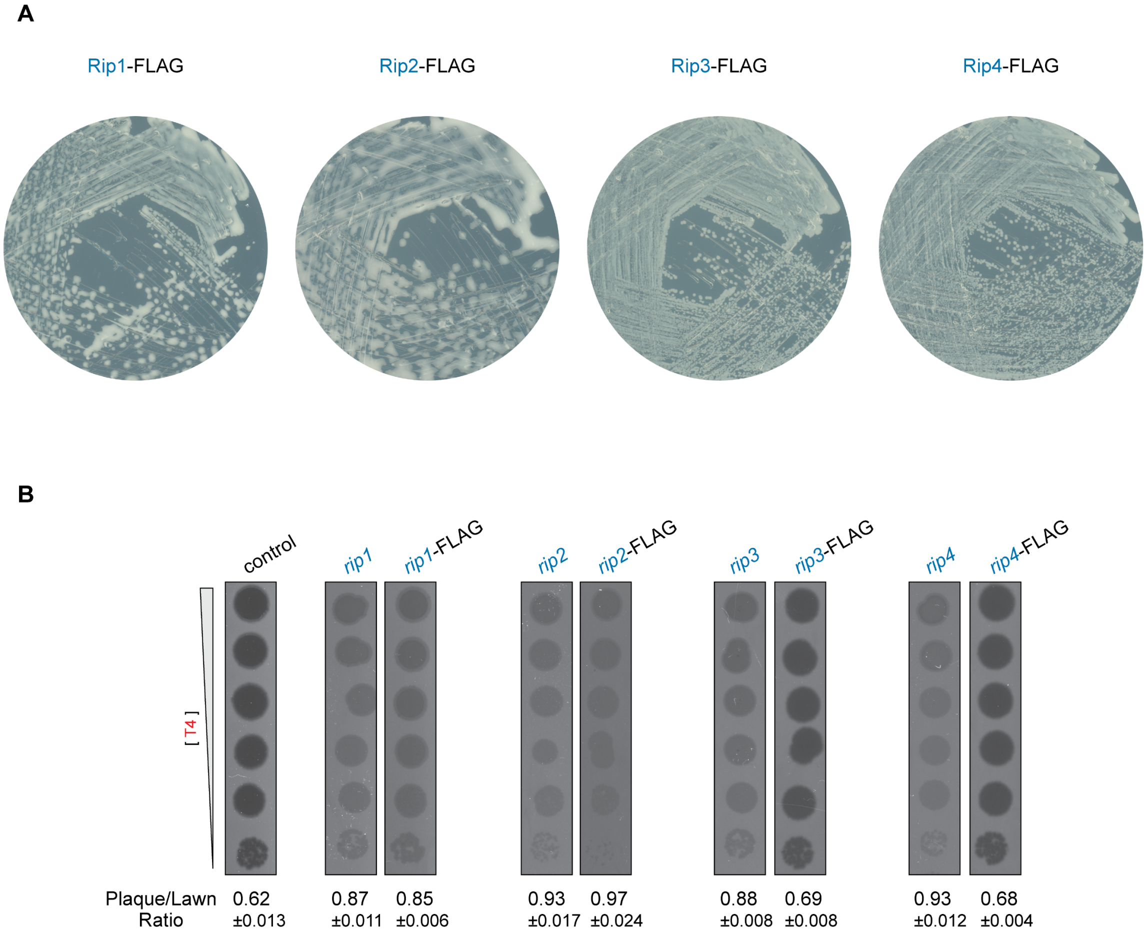

Figure S3 | Functional analysis of C-terminally FLAG-tagged Rip proteins

(A) Rip proteins were C-terminally tagged with a FLAG epitope and tested for their ability to induce mucoidy, similar to their untagged counterparts. Rip1 and Rip2 retained function, while Rip3 and Rip4 lost activity upon tagging.

(B) Phage plaque assay of T4 using tenfold serial dilutions on lawns of WT strains expressing either a control plasmid, untagged *rip*1–4, or C-terminally tagged versions of the Rip proteins.

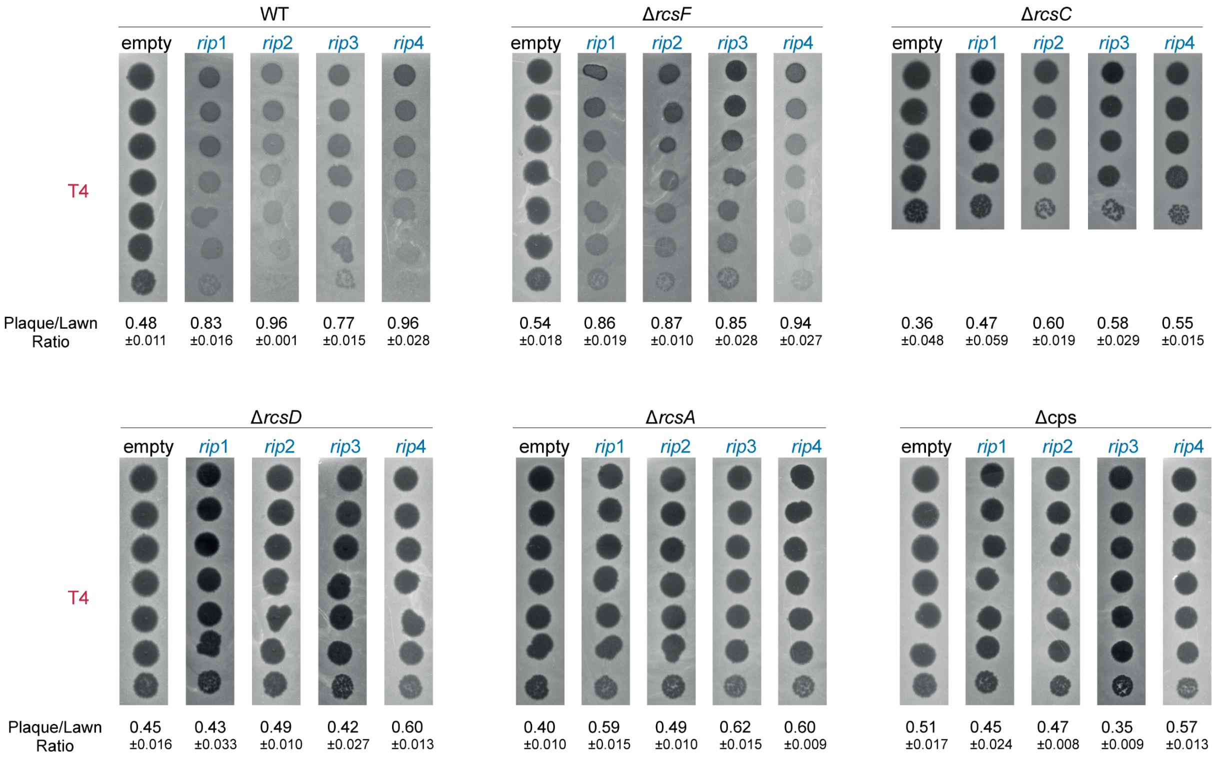

Figure S4 | Rcs pathway components required for Rip-mediated anti-phage defense

Phage plaque assay of T4 using tenfold serial dilutions on lawns of WT, Δ*rcsF*, Δ*rcsD*, Δ*rcsA*, or Δ*wca* strains expressing *rip1-4* or a control plasmid.

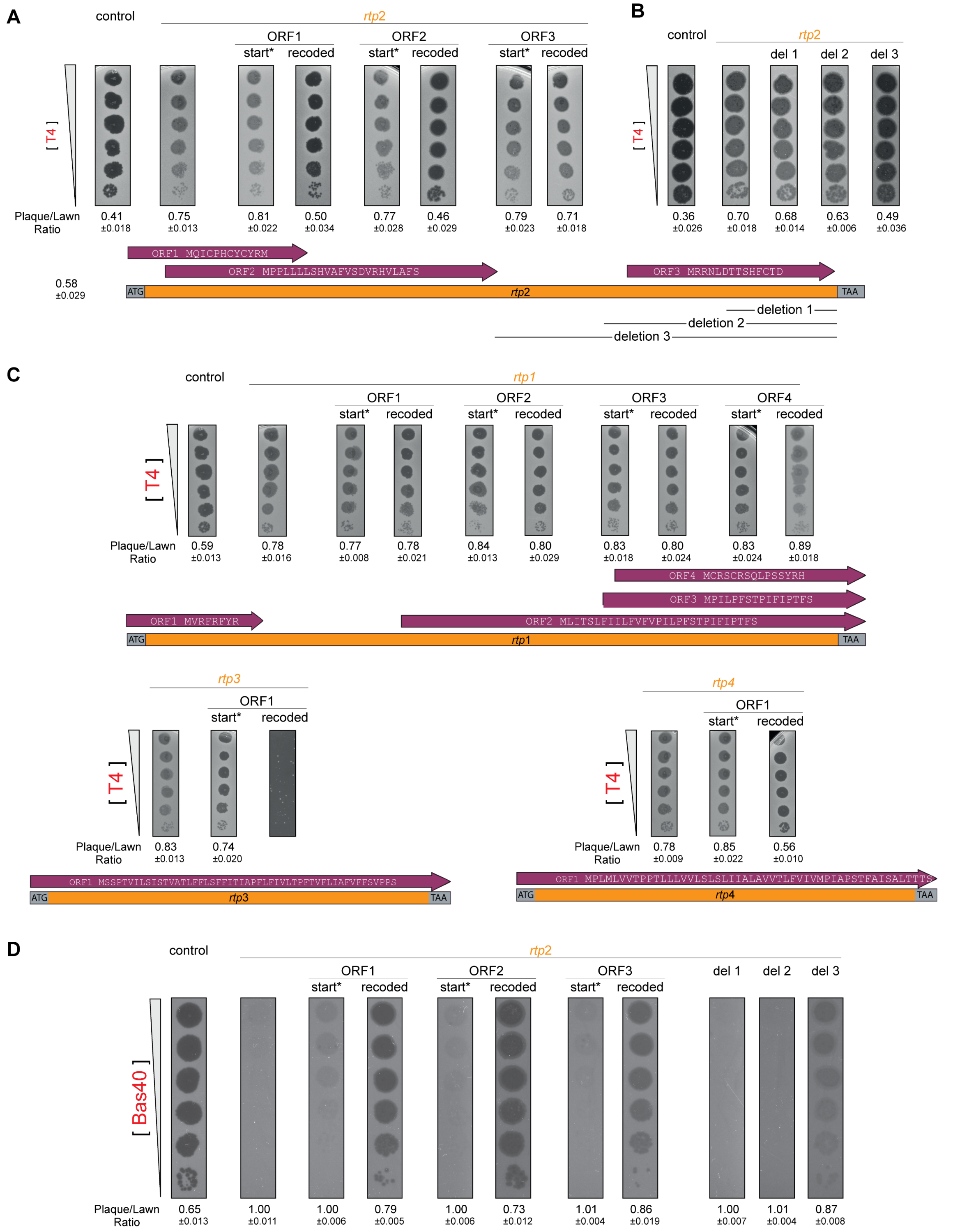

Figure S5 | Mutational analysis of *rtp1-4*

(A-C) T4 plaquing on cells producing the indicated *rtp* genes with the listed mutations. Plaque/lawn ratios are shown below each panel. These assays were

intended to test whether *rtp* genes function through translated ORFs or RNA elements using two strategies: (i) start codon mutations to block translation without altering RNA context, or (ii) extensive synonymous recoding to modify mRNA sequence while preserving protein sequence. For *rtp1*, both strategies preserved anti-phage activity, leaving its mechanism unresolved. rtp2 and *rtp4* maintained function after start codon mutations but lost activity following synonymous recoding, supporting an RNA-level mode of action. *rtp3* retained activity after start codon mutation, suggesting translation is dispensable, but its recoded variant was toxic, preventing assessment. Note: Figures S5A and S5C were performed together, and the same image of the control culture (cells expressing the empty vector) is shown in both panels for easier comparison.

(D) Phage plaque assay of Bas40 using tenfold serial dilutions on lawns of strains expressing *rtp2* variants or control plasmid.

```
rip-T4-1        ATG--GTTCGGTTCCGATT----CTACCGCTAACAGTAACCCTCATACTATCTCTCTCAA
rip-T4-4        ATG-----------CCACTGATGCTGGTGGTGACGCCTCCTACTCTGTTGC-----TCGT
rip-T4-2        ATGCAGATTTGCCCCCACTG---TTATTGCT--------ATCGCATGTAGC---------
rip-T4-3        ATG--TCTTCGCCAACAGTCATCTTATCGAT--------CTCCACTGTGGCAACCCTCTT
                ***             * *     *   * *              *

rip-T4-1        CATCGCTGTTAGTGTTAATAACATC-------------TCTGTTTATTATCCTTTTCGTT
rip-T4-4        TGTC-TTGTCACTGTCACTGATCATTGC----------ACTTGCGGTAGTTACACTCTTC
rip-T4-2        ---------TTTTGTTTCTGATGTGCGCCACGTTTTAGCTTTTTCATAACCCTCACCCTC
rip-T4-3        TTTC-CTGTCTTTTTTTATAACAATTGCTCCATT----CCTCTTCATAGTTCTAACTCCG
                            * *   * *                   *     *

rip-T4-1        TTTGTGCCGATCCT----GCCGTTCTCAA--------CTCCCATCTTCATACCGAC---A
rip-T4-4        GTTATAGTAATGCCTATTGCC-CCCTCAACAT-----TTGCTATTTCTGCCCTTACTACA
rip-T4-2        CCTAGTTTCATCTTTTGCGTCGCAATCTGGATACGACTTCTCACTTTTGTACTGACTA--
rip-T4-3        TTTACTGTTTTCCTTATCGCC----------------TTTGTATTTTTCTCCGTAC--CG
                  *       *       * *                 *   *  *     *  **

rip-T4-1        TTCTCTTAATAA
rip-T4-4        ACTTCGTAATAA
rip-T4-2        --GTCGTAATAA
rip-T4-3        CCTTCGTAATAA
```

Figure S6 | Sequence diversity among selected random genes

Multiple sequence alignment demonstrating sequence divergence among *rtp1-4* genes.

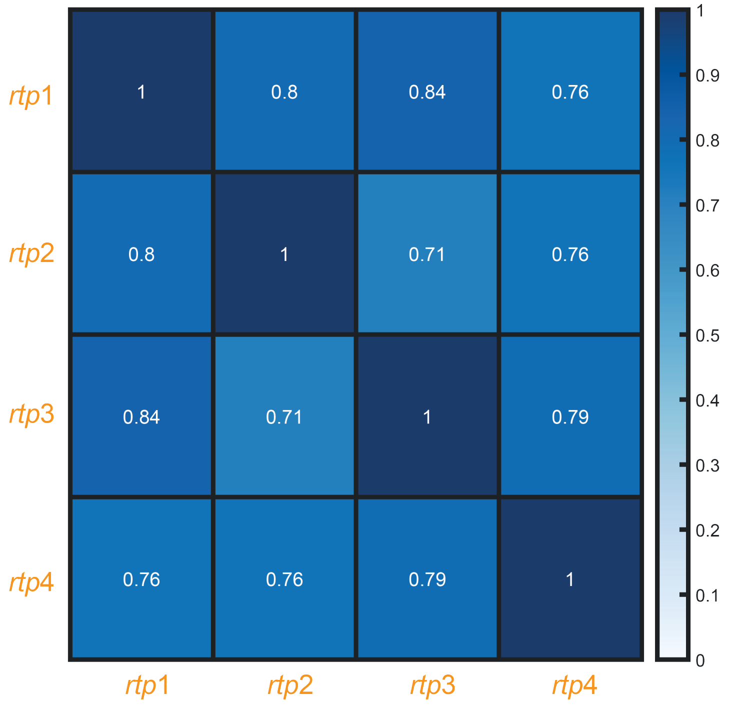

Figure S7 | Correlated transcriptional responses across *rtp*-expressing strains

Pairwise Pearson correlation coefficients of gene expression fold-changes relative to control cells reveal highly correlated transcriptional profiles among the four *rtp*-expressing strains, indicating a shared cellular response.

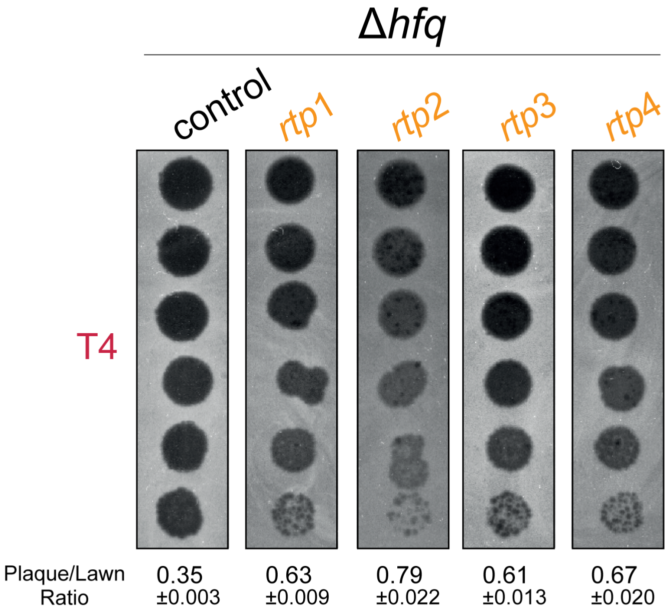

Figure S8 | *rtp1-4* maintain anti-phage defense in Δ*hfq* background

Phage plaque assay of T4 using tenfold serial dilutions on Δ*hfq* strains expressing *rtp1-4* or a control plasmid.

```
rtp2      ATGCAGATTTGCCCCCACTGTTATTGCTATCGC-----------ATGTAGCTTTTGTTTC
rtp1      ---ATGGTTCGGTTCCGATTCTACCGCTAACAGTAACCCTCATACTATCTCTCTCAACAT
rybB      -------------------GCCACTGCTTTTCT--------------------------T
rtp4      --------ATGCCACTGATGCTGGTGGTGACGCCTCCTACTCTGTTGCTCGTTGTCTTGT
malH      ----------------AAGGTTAAAGATGTTGT-----------TCTGCCAATGTTATGC
rtp3      --------ATGTCTTCGCCAACAGTCATCTTATCGATCTCCACTGTGGCAACCCTCTTTT
rseX      ------------------TTTTTATTATTCTGT-----------------------GTCA
micC      ---GTTATATGCCTTTATTGTCACAGATTTTAT-----------------------TTTC
                                     *

rtp2      TGATGTGCGCCACGTTTTAGCTTTTTCATAACCCTCACCC--TCCCTAGTTTCATCTTTT
rtp1      CGCTGT-------------TAGTGTTAATAACATCTCTGTTTATTATCCTTTTCGTTTTT
rybB      TGATGT-------------CCCCATTTTGTGGAGCCCATCAACCCCGCCATTTCGGTTCA
rtp4      CACTGT-------------CACTGATCATTGCACTTGCGGTAGTTACACTCTTCGTTATA
malH      CGCTGC-------------ACCCTCAACTTACGTTA------TCCCAACTTGTGACTGTT
rtp3      TCCTGT-------------CTTTTTTTATAACAATTGCTCCATTCCTCTTCATAGTTCTA
rseX      TGATGC-------------TTCCGTTATTAGCCTTTTATC--GTCTTGTTTATATTTTTT
micC      TGTTGG-------------GCCATTGCATTGCCACTGATT--TTCCAACATATAA----A
             **

rtp2      GCGTCG----CAATCTGGATACGACTTCTCACTTTTGTACTGACTA----GTCGTAATAA
rtp1      GTGCCG------ATCCTGCCGTTCTCAACTCCCATCTTCATACCGACATTCTCTTAATAA
rybB      AGGTTG---------------------ATGGGTTTTTT----------------------
rtp4      GTAATGCCTATTGCCCCCTCAACATTTGCTATTTCTGCCCTTACTACAACTTCGTAATAA
malH      ATTCGG-----CGCTCCACGGAGC------GCCTTTTTTT--------------------
rtp3      ACTCCGTTTACTGTTTTCCTTATCGCCTTTGTATTTTTCTCCGTACCGCCTTCGTAATAA
rseX      GGGCCG-------------GCATGATGCCGGCTTTTTTTT--------------------
micC      AAGACA-----AGCCCGAACAGTCGTCCGGGCTTTTTTTTT-------------------
```

Figure S9 | Comparison of *rtp1-4* genes with known OmpC-regulating sRNAs

Multiple sequence alignment comparing known OmpC-regulating sRNAs (RybB, MicC, RseX, and MicH) with *rtp1-4*.

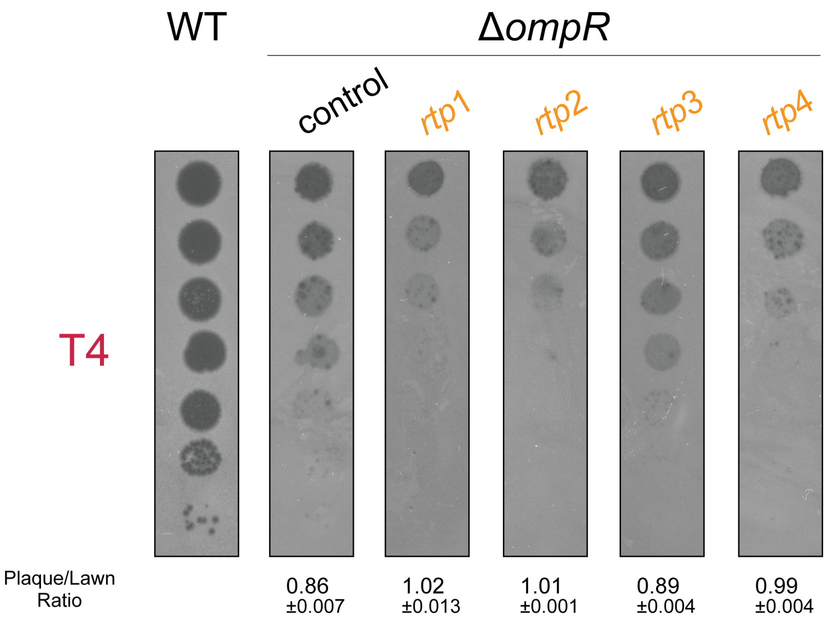

Figure S10 | *rtp1-4* anti-phage defense in Δ*ompR* background

Phage plaque assay of T4 using tenfold serial dilutions on Δ*ompR* strains expressing *rtp1-4* or a control plasmid.

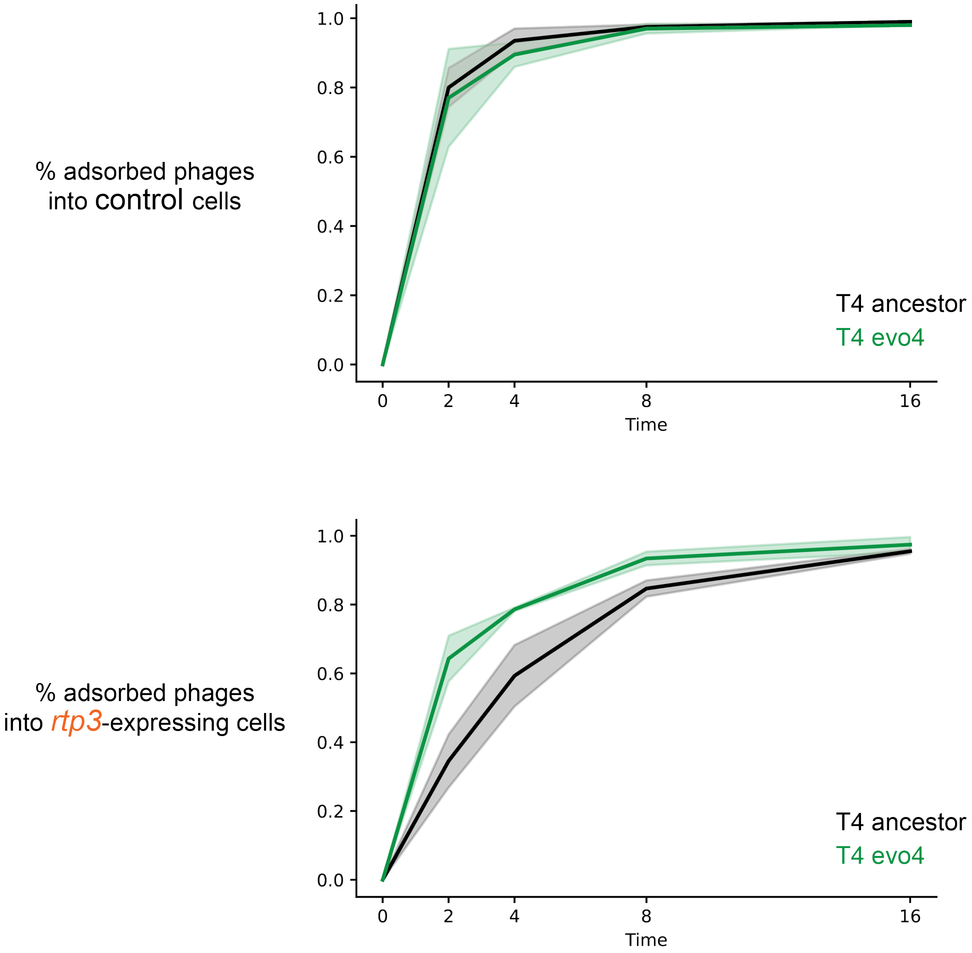

Figure S11 | Adsorption of ancestral and evo4 T4 phage on control and rtp3-expressing cells

Percentage of adsorbed T4 phages measured over time (0, 2, 4, 8, and 16 minutes post-infection), comparing the adsorption rates of ancestral and evo4 T4 phages on cells carrying either an empty control plasmid or expressing *rtp3*.

Supporting Information Dataset Legends

Dataset S1 (separate file). Key resources table. Strains, plasmids, antibodies, reagents, and other resources used in this study.

Dataset S2 (separate file). Mutations to T4 genomes following selection on strains expressing *rtp2*. Six independent selections and the corresponding mutations to the T4 genome are shown.

SI References

1. C. Welinder, L. Ekblad, Coomassie staining as loading control in Western blot analysis. *J Proteome Res* **10**, 1416–1419 (2011).
2. M. A. Dillies, *et al.*, A comprehensive evaluation of normalization methods for Illumina high-throughput RNA sequencing data analysis. *Brief Bioinform* **14**, 671–683 (2013).
3. J. Abramson, *et al.*, Accurate structure prediction of biomolecular interactions with AlphaFold 3. *Nature* **630**, 493–500 (2024).
